# Supplementary material for: In vitro immunoreactivity and in vivo neutralization of Trimeresurus gracilis venom with antivenoms targeting four pit viper species
Source: PLoS Negl Trop Dis. 2024 Mar 25;18(3):e0012070. doi: 10.1371/journal.pntd.0012070 (PMC10994551; doi:10.1371/journal.pntd.0012070)
Supplement: Attachment — Submitted filename: Ans_reviewer_20240311.docx [file pntd.0012070.s002.docx]

**Reviewer #1:**

The methods are justified and clearly described. The level of detail is enough to reproduce the results without havind to consult multiple references. The methods are addecuate to test the presented hypothesis and the results support the conclusions. The well-being of the snakes used in the study is granted and the number of animals used in the in vivo experiment is justified to reach the necessary statistical significance.

Reply: Thank you for your positive comments.

The calculation of Emax should be explained further as comparison accross plates in ELISA is difficult without an external control to normalize the absorbance intensity.

Reply: Using the four parameter logistic curve-fitting in SigmaPlot (version 12.0; SYSTAT Software Inc., CA, USA), the statistical values (mean, standard error, and 95% confidence interval) for E_max_ and EC_50_ were determined. We agree that an internal control will allow to normalize across plates and better compare the E_max_ values across plates, while it's true that numerous published articles conducting ELISA (referenced below) did not utilize an internal control. In our study, we have mitigated the impact of variation across ELISA plates by controlling experimental factors. This includes maintaining a consistent reaction time and temperature, using identical batch numbers for ELISA plates and the same ELISA reader, and ensuring the participation of the same experimenter. We have made revisions to the relevant text at Line 187-189, 259-262, 432-439.

References:

Alsolaiss, J., Alomran, N., Hawkins, L., & Casewell, N. R. (2023). Commercial Antivenoms Exert Broad Paraspecific Immunological Binding and In Vitro Inhibition of Medically Important Bothrops Pit Viper Venoms. Toxins 15(1), 1. https://doi.org/10.3390/toxins15010001

Sousa, L.F., Nicolau, C.A., Peixoto, P.S., Bernardoni, J.L., Oliveira, S.S., et al. (2013) Comparison of Phylogeny, Venom Composition and Neutralization by Antivenom in Diverse Species of Bothrops Complex. PLoS Negl Trop Dis 7(9): e2442. doi:10.1371/journal.pntd.0002442

Alomran, N.; Blundell, P.; Alsolaiss, J.; Crittenden, E.; Ainsworth, S.; Dawson, C.A.; Edge, R.J.; Hall, S.R.; Harrison, R.A.; Wilkinson, M.C.; et al. Exploring the Utility of Recombinant Snake Venom Serine Protease Toxins as Immunogens for Generating Experimental Snakebite Antivenoms. Toxins 2022, 14, 443. https://doi.org/10.3390/toxins14070443

Casewell, N. R., Al-Abdulla, I., Smith, D., Coxon, R., & Landon, J. (2014). Immunological cross-reactivity and neutralisation of European viper venoms with the monospecific Vipera berus antivenom ViperaTAb. Toxins 6(8), 2471–2482. https://doi.org/10.3390/toxins6082471

Chaisakul, J., Rusmili, M. R. A., Alsolaiss, J., Albulescu, L. O., Harrison, R. A., Othman, I., & Casewell, N. R. (2020). In Vitro Immunological Cross-Reactivity of Thai Polyvalent and Monovalent Antivenoms with Asian Viper Venoms. Toxins 12(12), 766. https://doi.org/10.3390/toxins12120766

Harrison, R. A., Oluoch, G. O., Ainsworth, S., Alsolaiss, J., Bolton, F., Arias, A. S., Gutiérrez, J. M., Rowley, P., Kalya, S., Ozwara, H., & Casewell, N. R. (2017). Preclinical antivenom-efficacy testing reveals potentially disturbing deficiencies of snakebite treatment capability in East Africa. PLoS neglected tropical diseases 11(10), e0005969. https://doi.org/10.1371/journal.pntd.0005969

Tan, C. H., Liew, J. L., Tan, N. H., Ismail, A. K., Maharani, T., Khomvilai, S., & Sitprija, V. (2017). Cross reactivity and lethality neutralization of venoms of Indonesian Trimeresurus complex species by Thai Green Pit Viper Antivenom. Toxicon 140, 32–37. https://doi.org/10.1016/j.toxicon.2017.10.014

The results confirm the hypothesis and support the conclusions. The results are clearly presented in a chronologicla manner that allos for the correct interpretation of the results. The tables are of sufficient quality. However, the figures (specially the ELISA results) can benefit of better resolution if the authors want to keep the snake photo on each corner. If that is the case, for consistency, please put the same snake photo in last two figures. Figure footnages are clear and don't require modifications-

Reply: Thank you and we have improved the resolution of the snake photos in the figures and put the same snake photo in last two figures.

The conclusions are supported by th data presented. The only limitation that I miss ackowledgment for is that the preincubation neutrlaisation experiment does not replicate a real envenomation case in which and it would be necessary a rescue experiment in which the venom is injected and then the antivenom is given. However, this is an excellent starting point to determine which antivenom to use that has immediate impact in the clinical treatment of envenomation. Public health relevance is addressed.

Reply: We acknowledge that rescue experiments more closely simulate real envenomation cases compared to preincubation neutralization experiments. However, it is worth noting that a majority of relevant studies (referenced below, except Tan et al., 2015) commonly employ the latter design. We believe preincubation neutralization experiments may directly test the efficacy of the serum. Additionally, we opted for preincubation neutralization experiments in our research to facilitate comparisons with other studies. Nonetheless, we recognize the importance of incorporating rescue experiments in future research. We have made revisions to the relevant text at Line 427-432.

References:

Alsolaiss, J., Alomran, N., Hawkins, L., & Casewell, N. R. (2023). Commercial Antivenoms Exert Broad Paraspecific Immunological Binding and In Vitro Inhibition of Medically Important Bothrops Pit Viper Venoms. Toxins 15(1), 1. https://doi.org/10.3390/toxins15010001

Sousa, L.F., Nicolau, C.A., Peixoto, P.S., Bernardoni, J.L., Oliveira, S.S., et al. (2013) Comparison of Phylogeny, Venom Composition and Neutralization by Antivenom in Diverse Species of Bothrops Complex. PLoS Negl Trop Dis 7(9): e2442. doi:10.1371/journal.pntd.0002442

Silva, G. M., Berto, D. H., Lima, C. A., Waitman, K. B., Lima, C. F. G., Prezoto, B. C., Vieira, M. L., Rocha, M. M. T., Gonçalves, L. R. C., & Andrade, S. A. (2021a). Synergistic effect of serine protease inhibitors and a bothropic antivenom in reducing local hemorrhage and coagulopathy caused by Bothrops jararaca venom. Toxicon 199, 87–93. https://doi.org/10.1016/j.toxicon.2021.06.009

Alomran, N.; Blundell, P.; Alsolaiss, J.; Crittenden, E.; Ainsworth, S.; Dawson, C.A.; Edge, R.J.; Hall, S.R.; Harrison, R.A.; Wilkinson, M.C.; et al. Exploring the Utility of Recombinant Snake Venom Serine Protease Toxins as Immunogens for Generating Experimental Snakebite Antivenoms. Toxins 2022, 14, 443. https://doi.org/10.3390/toxins14070443

Casewell, N. R., Al-Abdulla, I., Smith, D., Coxon, R., & Landon, J. (2014). Immunological cross-reactivity and neutralisation of European viper venoms with the monospecific Vipera berus antivenom ViperaTAb. Toxins 6(8), 2471–2482. https://doi.org/10.3390/toxins6082471

Tan, C. H., Tan, K. Y., Lim, S. E., & Tan, N. H. (2015). Venomics of the beaked sea snake, Hydrophis schistosus: A minimalist toxin arsenal and its cross-neutralization by heterologous antivenoms. Journal of proteomics 126, 121–130. https://doi.org/10.1016/j.jprot.2015.05.035

Leong, P. K., Tan, C. H., Sim, S. M., Fung, S. Y., Sumana, K., Sitprija, V., & Tan, N. H. (2014). Cross neutralization of common Southeast Asian viperid venoms by a Thai polyvalent snake antivenom (Hemato Polyvalent Snake Antivenom). Acta tropica 132, 7–14. https://doi.org/10.1016/j.actatropica.2013.12.015

Harrison, R. A., Oluoch, G. O., Ainsworth, S., Alsolaiss, J., Bolton, F., Arias, A. S., Gutiérrez, J. M., Rowley, P., Kalya, S., Ozwara, H., & Casewell, N. R. (2017). Preclinical antivenom-efficacy testing reveals potentially disturbing deficiencies of snakebite treatment capability in East Africa. PLoS neglected tropical diseases 11(10), e0005969. https://doi.org/10.1371/journal.pntd.0005969

Tan, C. H., Liew, J. L., Tan, N. H., Ismail, A. K., Maharani, T., Khomvilai, S., & Sitprija, V. (2017). Cross reactivity and lethality neutralization of venoms of Indonesian Trimeresurus complex species by Thai Green Pit Viper Antivenom. Toxicon 140, 32–37. https://doi.org/10.1016/j.toxicon.2017.10.014

Minor

• Line 52: As it is presented right now on the results and discussion, the findings do not offer a clear way to develop more effective antivenoms. I suggest that the authors include in the discussion a note about how this results can guide the development of cross-neutralizing monoclonal antibodies or the development of better cross-neutralizing formulations to keep such statement and improve the reach of the discussion

Reply: We have included an experiment involving SDS-PAGE analysis of *T. gracilis* venom, comparing it to other venoms in our study. This addition aims to enhance the discussion regarding antivenom binding capacity levels and improve our understanding of toxins that may not be recognized by traditional antivenoms. In an ongoing study investigating the binding capacity of TsPmAV to *T. gracilis* venom through immunoaffinity column chromatography, we observed lower reactivity with P-I SVMPs and certain SVSPs/PLA_2_ when exposed to the antivenom (Tsai et al., unpublished data). Silva et al. (2021a, b) discovered that the antivenom targeting *Bothrops* venom has limitations in addressing local effects such as hemorrhage and neutralizing SVSPs. However, they observed that supplementing the antivenom with a serine protease inhibitor can enhance its overall efficacy. In subsequent studies, testing the neutralization efficacy by adding inhibitors specific to P-I SVMPs or SVSPs/PLA_2_ to the existing antivenom could be explored to enhance therapeutic success for both local and systemic symptoms occurred in *T. gracilis* envenomation. We have made revisions to the relevant text at Line 33-36, 112-114, 169-174, 200-209, 282-307, 395-420, 452-459.

References:

Silva, G. M., Berto, D. H., Lima, C. A., Waitman, K. B., Lima, C. F. G., Prezoto, B. C., Vieira, M. L., Rocha, M. M. T., Gonçalves, L. R. C., & Andrade, S. A. (2021a). Synergistic effect of serine protease inhibitors and a bothropic antivenom in reducing local hemorrhage and coagulopathy caused by *Bothrops jararaca* venom. Toxicon 199, 87–93. https://doi.org/10.1016/j.toxicon.2021.06.009

Silva, G. M., de Souza, D. H. B., Waitman, K. B., Ebram, M. C., Fessel, M. R., Zainescu, I. C., Portaro, F. C., Heras, M., & de Andrade, S. A. (2021b). Design, synthesis, and evaluation of *Bothrops* venom serine protease peptidic inhibitors. The Journal of Venomous Animals and Toxins Including Tropical Diseases 27, e20200066. https://doi.org/10.1590/1678-9199-JVATITD-2020-0066

• Line 177: Why was the mixture incubated at 37C?

Reply: Many studies on snake venom neutralization, as referenced below, commonly incubated the reaction mixture at 37°C (reflecting the body temperature of mammals such as rodents). To ensure consistency and facilitate result comparisons among related studies, we adopted the same temperature setting for the preincubation neutralization experiment in our research.

References:

Alsolaiss, J., Alomran, N., Hawkins, L., & Casewell, N. R. (2023). Commercial Antivenoms Exert Broad Paraspecific Immunological Binding and In Vitro Inhibition of Medically Important Bothrops Pit Viper Venoms. Toxins 15(1), 1. https://doi.org/10.3390/toxins15010001

Sousa, L.F., Nicolau, C.A., Peixoto, P.S., Bernardoni, J.L., Oliveira, S.S., et al. (2013) Comparison of Phylogeny, Venom Composition and Neutralization by Antivenom in Diverse Species of Bothrops Complex. PLoS Negl Trop Dis 7(9): e2442. doi:10.1371/journal.pntd.0002442

Silva, G. M., Berto, D. H., Lima, C. A., Waitman, K. B., Lima, C. F. G., Prezoto, B. C., Vieira, M. L., Rocha, M. M. T., Gonçalves, L. R. C., & Andrade, S. A. (2021a). Synergistic effect of serine protease inhibitors and a bothropic antivenom in reducing local hemorrhage and coagulopathy caused by Bothrops jararaca venom. Toxicon 199, 87–93. https://doi.org/10.1016/j.toxicon.2021.06.009

Alomran, N.; Blundell, P.; Alsolaiss, J.; Crittenden, E.; Ainsworth, S.; Dawson, C.A.; Edge, R.J.; Hall, S.R.; Harrison, R.A.; Wilkinson, M.C.; et al. Exploring the Utility of Recombinant Snake Venom Serine Protease Toxins as Immunogens for Generating Experimental Snakebite Antivenoms. Toxins 2022, 14, 443. https://doi.org/10.3390/toxins14070443

Casewell, N. R., Al-Abdulla, I., Smith, D., Coxon, R., & Landon, J. (2014). Immunological cross-reactivity and neutralisation of European viper venoms with the monospecific Vipera berus antivenom ViperaTAb. Toxins 6(8), 2471–2482. https://doi.org/10.3390/toxins6082471

Leong, P. K., Tan, C. H., Sim, S. M., Fung, S. Y., Sumana, K., Sitprija, V., & Tan, N. H. (2014). Cross neutralization of common Southeast Asian viperid venoms by a Thai polyvalent snake antivenom (Hemato Polyvalent Snake Antivenom). Acta tropica 132, 7–14. https://doi.org/10.1016/j.actatropica.2013.12.015

Harrison, R. A., Oluoch, G. O., Ainsworth, S., Alsolaiss, J., Bolton, F., Arias, A. S., Gutiérrez, J. M., Rowley, P., Kalya, S., Ozwara, H., & Casewell, N. R. (2017). Preclinical antivenom-efficacy testing reveals potentially disturbing deficiencies of snakebite treatment capability in East Africa. PLoS neglected tropical diseases 11(10), e0005969. https://doi.org/10.1371/journal.pntd.0005969

Tan, C. H., Liew, J. L., Tan, N. H., Ismail, A. K., Maharani, T., Khomvilai, S., & Sitprija, V. (2017). Cross reactivity and lethality neutralization of venoms of Indonesian Trimeresurus complex species by Thai Green Pit Viper Antivenom. Toxicon 140, 32–37. https://doi.org/10.1016/j.toxicon.2017.10.014

• Preincubation but not rescue

Reply: We acknowledge that rescue experiments more closely simulate real envenomation cases compared to preincubation neutralization experiments. However, it is worth noting that a majority of relevant studies commonly employ the latter design. We believe preincubation neutralization experiments may directly test the efficacy of the serum. Additionally, we opted for preincubation neutralization experiments in our research to facilitate comparisons with other studies. Nonetheless, we recognize the importance of incorporating rescue experiments in future research. We have made revisions to the relevant text at Line 427-432.

• Table 1: T. Stejnegeri Dry venom yield (mg): 4.60 - keep significant ciphers throughout the table

Reply: We have revised Table 1.

• The ELISA values can vary a lot depending on the time left for the assay to reveal. Without a internal control that allow to normalize across plates, Emax value cannot be compared across plates. Also, they are close to saturation of absorbance signal (which usually occurs around 2.0 absorbance units).

Reply: We agree that an internal control will allow to normalize across plates and better compare the E_max_ values across plates, while it's true that numerous published articles conducting ELISA did not utilize an internal control. In our study, we have mitigated the impact of variation across ELISA plates by controlling experimental factors. This includes maintaining a consistent reaction time and temperature, using identical batch numbers for ELISA plates and the same ELISA reader, and ensuring the participation of the same experimenter. We have made revisions to the relevant text at Line 432-439. Furthermore, it is worth mentioning that absorbance values exceeding 2.0 or 3.0, as observed in various studies (referenced below), are not uncommon.

References:

Alomran, N.; Blundell, P.; Alsolaiss, J.; Crittenden, E.; Ainsworth, S.; Dawson, C.A.; Edge, R.J.; Hall, S.R.; Harrison, R.A.; Wilkinson, M.C.; et al. Exploring the Utility of Recombinant Snake Venom Serine Protease Toxins as Immunogens for Generating Experimental Snakebite Antivenoms. Toxins 2022, 14, 443. https://doi.org/10.3390/toxins14070443

Chaisakul, J., Rusmili, M. R. A., Alsolaiss, J., Albulescu, L. O., Harrison, R. A., Othman, I., & Casewell, N. R. (2020). In Vitro Immunological Cross-Reactivity of Thai Polyvalent and Monovalent Antivenoms with Asian Viper Venoms. Toxins 12(12), 766. https://doi.org/10.3390/toxins12120766

Harrison, R. A., Oluoch, G. O., Ainsworth, S., Alsolaiss, J., Bolton, F., Arias, A. S., Gutiérrez, J. M., Rowley, P., Kalya, S., Ozwara, H., & Casewell, N. R. (2017). Preclinical antivenom-efficacy testing reveals potentially disturbing deficiencies of snakebite treatment capability in East Africa. PLoS neglected tropical diseases 11(10), e0005969. https://doi.org/10.1371/journal.pntd.0005969

Tan, C. H., Liew, J. L., Tan, N. H., Ismail, A. K., Maharani, T., Khomvilai, S., & Sitprija, V. (2017). Cross reactivity and lethality neutralization of venoms of Indonesian Trimeresurus complex species by Thai Green Pit Viper Antivenom. Toxicon 140, 32–37. https://doi.org/10.1016/j.toxicon.2017.10.014

• Line 261: T.gracilisvenom (add space between)

Reply: We amended accordingly (Line 334).

**Reviewer #2:**

-Are the objectives of the study clearly articulated with a clear testable hypothesis stated?

Yes, the objectives of the study "In vitro and in vivo cross-neutralization of Trimeresurus gracilis venom using antivenoms toward four pit viper species" is articulated with a testable hypothesis. This study contributes to reinforce the importance of detailed and in-depth knowledge of the composition of venoms for the development of effective antivenoms on the treatment of snakebite. In addition, this article shows that in 2024, there are no specific antivenoms for some species of snakes of medical importance. However, the authors with data available in the literature could better discuss about the venoms’ composition, about toxins, such as serine proteases from the venom of snakes of the genus Bothrops, which are known not to be totally neutralized by their specific antivenom, and which are involved in clinical aspects of Bothropic envenomation, such as coagulopathy, as observed by the authors of this article. “However, only two case reports available to date indicate that patients bitten on the finger by this species, not only experience local swelling, bruising, hemorrhagic blisters, or tissue necrosis but also systemic symptoms such as coagulopathy”.

Reply: We have referred to the venomic and antivenomic studies of snakes of the genus *Bothrops*. Commercial antivenoms may exert broad paraspecific immunological binding and neutralization of medically important snake venoms, as observed in *Bothrops* (Alsolaiss et al., 2023). Sousa et al. (2013) compared the composition and reactivity with multispecific *Bothrops* antivenom (SAB) of venoms collected from six species of *Bothrops*, *Bothropoides* and *Rhinocerophis* snakes, and found that P-III snake venom metalloproteinases (SVMPs) are the most antigenic toxins in the venoms of snakes from the *Bothrops* complex, while class P-I SVMPs, snake venom serine proteinases (SVSPs) and phospholipases A_2_ (PLA_2_) reacted with SAB in lower levels. We also have included an experiment involving SDS-PAGE analysis of *T. gracilis* venom, comparing it to other venoms in our study. This addition aims to enhance the discussion regarding antivenom binding capacity levels and improve our understanding of toxins that may not be recognized by traditional antivenoms. In an ongoing study investigating the binding capacity of TsPmAV to *T. gracilis* venom through immunoaffinity column chromatography, we observed lower reactivity with P-I SVMPs and certain SVSPs/PLA_2_ when exposed to the antivenom (Tsai et al., unpublished data). Silva et al. (2021a, b) discovered that the antivenom targeting *Bothrops* venom has limitations in addressing local effects such as hemorrhage and neutralizing SVSPs. However, they observed that supplementing the antivenom with a serine protease inhibitor can enhance its overall efficacy. In subsequent studies, testing the neutralization efficacy by adding inhibitors specific to P-I SVMPs or SVSPs/PLA_2_ to the existing antivenom could be explored to enhance therapeutic success for both local and systemic symptoms. We have made revisions to the relevant text at Line 33-36, 112-114, 169-174, 200-209, 282-307, 395-420, 452-459.

References:

Alsolaiss, J., Alomran, N., Hawkins, L., & Casewell, N. R. (2023). Commercial Antivenoms Exert Broad Paraspecific Immunological Binding and In Vitro Inhibition of Medically Important Bothrops Pit Viper Venoms. Toxins 15(1), 1. https://doi.org/10.3390/toxins15010001

Sousa, L.F., Nicolau, C.A., Peixoto, P.S., Bernardoni, J.L., Oliveira, S.S., et al. (2013) Comparison of Phylogeny, Venom Composition and Neutralization by Antivenom in Diverse Species of Bothrops Complex. PLoS Negl Trop Dis 7(9): e2442. doi:10.1371/journal.pntd.0002442

Silva, G. M., Berto, D. H., Lima, C. A., Waitman, K. B., Lima, C. F. G., Prezoto, B. C., Vieira, M. L., Rocha, M. M. T., Gonçalves, L. R. C., & Andrade, S. A. (2021a). Synergistic effect of serine protease inhibitors and a bothropic antivenom in reducing local hemorrhage and coagulopathy caused by Bothrops jararaca venom. Toxicon 199, 87–93. https://doi.org/10.1016/j.toxicon.2021.06.009

Silva, G. M., de Souza, D. H. B., Waitman, K. B., Ebram, M. C., Fessel, M. R., Zainescu, I. C., Portaro, F. C., Heras, M., & de Andrade, S. A. (2021b). Design, synthesis, and evaluation of *Bothrops* venom serine protease peptidic inhibitors. The Journal of Venomous Animals and Toxins Including Tropical Diseases 27, e20200066. https://doi.org/10.1590/1678-9199-JVATITD-2020-0066

-Is the study design appropriate to address the stated objectives?

Yes, the study design is appropriate to address the objectives. However, experiments, such as an SDS-PAGE of the Trimeresurus gracilis venom compared to the other venoms studied here, could help in the understanding of the differences in compositions. For these reasons, I strongly suggest that the authors do this analysis.

The authors could correlate electrophoresis data and associate with the literature to improve the discussion about the levels of antivenoms binding capacity and knowledge about the toxins that are not recognize by traditional antivenoms.

Besides this, another experiment, as western blotting could improve the antivenoms binding capacity comprehension. However, the authors mentioned that the problem about the antivenoms quantity.

Reply: We have included an experiment involving SDS-PAGE analysis of *T. gracilis* venom, comparing it to other venoms in our study. This addition aims to enhance the discussion regarding antivenom binding capacity levels and improve our understanding of toxins that may not be recognized by traditional antivenoms. We opted not to conduct western blotting partially due to concerns about the quantity of antivenoms. In our future studies, we plan to further investigate the compositions of *T. gracilis* venom neutralized by different antivenoms using western blotting and/or immunoaffinity column chromatography. We have made revisions to the relevant text at Line 33-36, 112-114, 169-174, 200-209, 282-307, 395-420, 452-459.

-Is the population clearly described and appropriate for the hypothesis being tested?

Yes, the population described the hypothesis tested. Characteristics, such as number, sex, size and reproductive status of each snake were described.

Reply: Thank you.

-Is the sample size sufficient to ensure adequate power to address the hypothesis being tested?

Considering the difficulties and ethical issues in the use of animals, the sample size is sufficient to ensure adequate to address the hypothesis.

Reply: Thank you.

-Were correct statistical analysis used to support conclusions?

Statistical analysis is unclear. Authors should describe better and in more detail the method used and the differences observed.

Reply: Using the four parameter logistic curve-fitting in SigmaPlot (version 12.0; SYSTAT Software Inc., CA, USA), the statistical values (mean, standard error, and 95% confidence interval) for E_max_ and EC_50_ were determined. As the venom samples from each species are pooled from individual snakes, we refrained from testing differences among treatments using t-tests, ANOVA, or nonparametric statistics, as observed in other studies (as referenced below). We have made revisions to the relevant text at Line 187-189, 259-262, 439-443.

References:

Alsolaiss, J., Alomran, N., Hawkins, L., & Casewell, N. R. (2023). Commercial Antivenoms Exert Broad Paraspecific Immunological Binding and In Vitro Inhibition of Medically Important Bothrops Pit Viper Venoms. Toxins 15(1), 1. https://doi.org/10.3390/toxins15010001

Alomran, N.; Blundell, P.; Alsolaiss, J.; Crittenden, E.; Ainsworth, S.; Dawson, C.A.; Edge, R.J.; Hall, S.R.; Harrison, R.A.; Wilkinson, M.C.; et al. Exploring the Utility of Recombinant Snake Venom Serine Protease Toxins as Immunogens for Generating Experimental Snakebite Antivenoms. Toxins 2022, 14, 443. https://doi.org/10.3390/toxins14070443

Chaisakul, J., Rusmili, M. R. A., Alsolaiss, J., Albulescu, L. O., Harrison, R. A., Othman, I., & Casewell, N. R. (2020). In Vitro Immunological Cross-Reactivity of Thai Polyvalent and Monovalent Antivenoms with Asian Viper Venoms. Toxins, 12(12), 766. https://doi.org/10.3390/toxins12120766

Tan, C. H., Tan, K. Y., Lim, S. E., & Tan, N. H. (2015). Venomics of the beaked sea snake, Hydrophis schistosus: A minimalist toxin arsenal and its cross-neutralization by heterologous antivenoms. Journal of proteomics 126, 121–130. https://doi.org/10.1016/j.jprot.2015.05.035

Leong, P. K., Tan, C. H., Sim, S. M., Fung, S. Y., Sumana, K., Sitprija, V., & Tan, N. H. (2014). Cross neutralization of common Southeast Asian viperid venoms by a Thai polyvalent snake antivenom (Hemato Polyvalent Snake Antivenom). Acta tropica 132, 7–14. https://doi.org/10.1016/j.actatropica.2013.12.015

Harrison, R. A., Oluoch, G. O., Ainsworth, S., Alsolaiss, J., Bolton, F., Arias, A. S., Gutiérrez, J. M., Rowley, P., Kalya, S., Ozwara, H., & Casewell, N. R. (2017). Preclinical antivenom-efficacy testing reveals potentially disturbing deficiencies of snakebite treatment capability in East Africa. PLoS neglected tropical diseases 11(10), e0005969. https://doi.org/10.1371/journal.pntd.0005969

Tan, C. H., Liew, J. L., Tan, N. H., Ismail, A. K., Maharani, T., Khomvilai, S., & Sitprija, V. (2017). Cross reactivity and lethality neutralization of venoms of Indonesian Trimeresurus complex species by Thai Green Pit Viper Antivenom. Toxicon 140, 32–37. https://doi.org/10.1016/j.toxicon.2017.10.014

-Does the analysis presented match the analysis plan?

Due to the lack of serum specific for treatment of envenomation by T. gracilis, victims are currently treated using a bivalent antivenom developed against T. stejnegeri and P. mucrosquamatus. This antivenom has limited effectiveness to treat the local effects. So, the authors investigated the cross-neutralization of T. gracilis venom using the bivalent antivenom. The results indicated that the bivalent antivenom was not completely effective in neutralizing T. gracilis venom.

Reply: Thank you.

-Are the results clearly and completely presented?

Yes, the results are clear. However, an electrophoresis analysis is necessary. I also suggest that the next lethality tests be delineated by a way that is closer to the reality of the accident. This could increase the correlation between the binding capacity of antivenoms and the efficacy of protection against the lethality caused by envenomation.

Reply: We have included an experiment involving SDS-PAGE analysis of *T. gracilis* venom, comparing it to other venoms in our study. This addition aims to enhance the discussion regarding antivenom binding capacity levels and improve our understanding of toxins that may not be recognized by traditional antivenoms. In our future studies, we plan to further investigate the compositions of *T. gracilis* venom neutralized by different antivenoms using western blotting and/or immunoaffinity column chromatography. We have made revisions to the relevant text at Line 33-36, 112-114, 169-174, 200-209, 282-307, 395-420, 452-459.

Also, the term "cross-neutralizing" should be replaced by "binding capacity", since the ELISA assay is an experiment of evaluating binding and not neutralizing activity.

Reply: We have made revisions to the relevant terms at Line 1, 33, 53, 110, 113, 226, 234, 378, 383, 390, 400, 405, 447, 449.

-Are the figures (Tables, Images) of sufficient quality for clarity?

Yes, the figures and images showed good quality.

Reply: Thank you.

-Are the conclusions supported by the data presented?

Yes, the data demonstrated that the bivalent antivenom against T. stejnegeri and P. mucrosquamatus is partially effective in neutralizing the venom of T. gracilis.

Reply: Thank you.

-Are the limitations of analysis clearly described?

Yes, but the authors should describe better and in more detail the statistical analysis.

Reply: Using the four parameter logistic curve-fitting in SigmaPlot (version 12.0; SYSTAT Software Inc., CA, USA), the statistical values (mean, standard error, and 95% confidence interval) for E_max_ and EC_50_ were determined. As the venom samples from each species are pooled from individual snakes, we refrained from testing differences among treatments using t-tests, ANOVA, or nonparametric statistics, as observed in other studies (as referenced below). We have described the limitations of our study at Line 439-443 and made revisions to the relevant text at Line 187-189, 259-262.

-Do the authors discuss how these data can be helpful to advance our understanding of the topic under study?

Yes, next studies should continue to identify the components of T. gracilis venom that are not neutralized by bivalent antivenom, which will be crucial for improving cross-neutralization treatments for envenomation by this snake.

Reply: Thank you.

-Is public health relevance addressed?

Yes. This study contributes to reinforce the importance of detailed and in-depth knowledge of the composition of venoms for the development of effective antivenoms on the treatment of snakebites. In addition, this article shows that in 2024, there are no specific antivenoms for some species of snakes of medical importance. These findings provide a reference for developing clinical treatment protocols and contribute to phylogenetic studies.

Reply: Thank you.

The article "In vitro and in vivo cross-neutralization of Trimeresurus gracilis venom using antivenoms toward four pit viper species" contributes to reinforce the importance of detailed and in-depth knowledge of the composition of venoms for the development of effective antivenoms. In addition, this article shows that in 2024, there are no specific antivenoms for some species of snakes of medical importance. However, the authors with data available in the literature could better discuss about the venoms’ composition, about toxins, such as serine proteases from the venom of snakes of the genus Bothrops, which are known not to be totally neutralized by their specific antivenom, and which are involved in clinical aspects of Bothropic envenomation, such as coagulopathy, as observed by the authors of this article. Experiments, such as an SDS-PAGE of the Trimeresurus gracilis venom compared to the other venoms studied here, could help in the understanding of the differences in compositions and the authors could correlate electrophoresis data and associate with the literature to improve the discussion about the levels of antivenoms binding capacity and knowledge about the toxins that are not recognize by traditional antivenoms. The bivalent antivenom was not completely effective in neutralizing T. gracilis venom. So, to identify the components of T. gracilis venom that are not neutralized by bivalent antivenom, will be crucial for improving the cross-neutralization and the development of the specific and effective antivenom to treat the victims of this snake.

Reply: We have referred the venomic and antivenomic studies of snakes of the genus *Bothrops*. Commercial antivenoms may exert broad paraspecific immunological binding and neutralization of medically important snake venoms, as observed in *Bothrops* (Alsolaiss et al., 2023). Sousa et al. (2013) compared the composition and reactivity with multispecific *Bothrops* antivenom (SAB) of venoms collected from six species of *Bothrops*, *Bothropoides* and *Rhinocerophis* snakes, and found that P-III snake venom metalloproteinases (SVMPs) are the most antigenic toxins in the venoms of snakes from the *Bothrops* complex, while class P-I SVMPs, snake venom serine proteinases (SVSPs) and phospholipases A_2_ (PLA_2_) reacted with SAB in lower levels. We also have included an experiment involving SDS-PAGE analysis of *T. gracilis* venom, comparing it to other venoms in our study. This addition aims to enhance the discussion regarding antivenom binding capacity levels and improve our understanding of toxins that may not be recognized by traditional antivenoms. In an ongoing study investigating the binding capacity of TsPmAV to *T. gracilis* venom through immunoaffinity column chromatography, we observed lower reactivity with P-I SVMPs and certain SVSPs/PLA_2_ when exposed to the antivenom (Tsai et al., unpublished data). Silva et al. (2021a, b) discovered that the antivenom targeting *Bothrops* venom has limitations in addressing local effects such as hemorrhage and neutralizing SVSPs. However, they observed that supplementing the antivenom with a serine protease inhibitor can enhance its overall efficacy. In subsequent studies, testing the neutralization efficacy by adding inhibitors specific to P-I SVMPs or SVSPs/PLA_2_ to the existing antivenom could be explored to enhance therapeutic success for both local and systemic symptoms. We have made revisions to the relevant text at Line 33-36, 112-114, 169-174, 200-209, 282-307, 395-420, 452-459.

Therefore, I recommend to accept this article with minor revision.

Reply: Thank you.

**Reviewer #3:**

The study proposed by Chuang and colleagues is quite interesting, concise, and objective, demonstrating cross-neutralization of available antivenoms for treating poisonings in Taiwan.

Reply: Thank you.

I have some suggestions to enhance the quality of the work:

1. In the study's introduction, the authors could highlight the epidemiology of these envenomings in Taiwan, including the number of cases for each species investigated in the study, mortality rates, sequelae, etc.

Reply: In Taiwan, majority (73.3%) of envenomation events were associated with *Trimeresurus stejnegeri* and *Protobothrops mucrosquamatus*, with case-fatality rates of 0.09%, during 2002–2014 based on the use of snake antivenoms (Hsu et al. 2023). Among Asian countries, Taiwan reported low incidence and case-fatality rates of snakebite envenomation, which might be due to easy access to modern medicine, immediate availability of antivenoms in healthcare facilities, few adverse effects of antivenoms, and prompt surgical intervention. Other crotaline species in Taiwan include *Deinagkistrodon acutus*, *Ovophis makazayazaya*, and *T. gracilis*. Only 0.6% of the envenomation events were associated with *D. acutus* (Hsu et al. 2023). As of now, there have been only two case reports of envenomations by *O. makazayazaya* (Chen et al. 2021; Luo et al. 2022) and two case reports of envenomations by *T. gracilis* in Taiwan (Tsai et al. 2022). The main effects of *T. stejnegeri* and *P. mucrosquamatus* bites in Taiwan were tissue swelling, pain, and local ecchymosis. In *T. stejnegeri* envenomation, bullae formation and coagulation function are generally normal, and patients can be administered one to two vials of the specific bivalent hemotoxic antivenom initially. In *P. mucrosquamatus* envenomation, bullae or necrosis is present, but coagulopathy and thrombocytopenia are uncommon. Patients can be administered two to four vials of the bivalent antivenom initially and the median total dose of the specific antivenom was 5.5 vials (Chiang et al. 2020; Mao et al. 2021; Mao and Hung 2015; Tsai et al. 2022; Hsu et al. 2023). *Deinagkistrodon acutus* envenomation is usually associated with hemorrhagic bullae or necrosis, prolonged PT/activated partial thromboplastin time, thrombocytopenia, and patients can be administered two to four vials of the monovalent antivenom initially (Mao and Hung 2015; Tsai et al. 2022; Su et al. 2018; Hsu et al. 2023; Cheng et al. 2017). In *O. makazayazaya* envenomation, patients may exhibit low fibrinogen and elevated D-dimer levels, and treatment may be initiated with one vial and completed with a total of five vials of the bivalent antivenom (Chen et al. 2021; Luo et al. 2022). We have made revisions to the relevant text at Line 68-87.

References:

Hsu, J.-Y., Chiang, S.-O., Yang, C.-C., Hsieh, T.W., Chung, C.-J., Mao, Y.-C. (2023) Nationwide and long-term epidemiological research of snakebite envenomation in Taiwan during 2002–2014 based on the use of snake antivenoms: A study utilizing National Health Insurance Database. PLoS Negl Trop Dis 17(6): e0011376. https://doi.org/10.1371/journal.pntd.0011376

Tsai, T. S., Chan, Y. Y., Huang, S. M., & Chuang, P. C. (2022). Case Report: Symptoms and Prognosis of *Trimeresurus gracilis* Envenomation. The American journal of tropical medicine and hygiene 106(4), 1281–1284. https://doi.org/10.4269/ajtmh.21-1144

Chen, M. H., Wu, S. H., Chen, Y. W., Lee, Y. L., & Chen, Y. C. (2021). A case report of *Ovophis makazayazaya* envenoming. Clinical toxicology (Philadelphia, Pa.) 59(7), 679–680. https://doi.org/10.1080/15563650.2020.1842666

Luo, Y., Mao, Y. C., Liu, P. Y., Chiang, L. C., Lai, C. S., Lin, W. L., & Huang, C. C. (2022). Case Report: Management of an Uncommon Crotaline Snakebite (*Ovophis makazayazaya*). The American journal of tropical medicine and hygiene 107(3), 705–708. Advance online publication. https://doi.org/10.4269/ajtmh.22-0255

Mao, Y.C., Hung, D.Z. 2015. Epidemiology of snake envenomation in Taiwan. Gopalakrishnakone, P., Faiz, M.A., Fernando, R., Gnanathasan, C.A., Habib, A.G., Yang, C.C., eds. Clinical Toxinology in Asia Pacific and Africa. Singapore: Spring. 2015:3-22.

Chiang, L. C., Tsai, W. J., Liu, P. Y., Ho, C. H., Su, H. Y., Lai, C. S., Lai, K. L., Lin, W. L., Lee, C. H., Yang, Y. Y., Doan, U. V., Maharani, T., & Mao, Y. C. (2020). Envenomation by Trimeresurus stejnegeri stejnegeri: clinical manifestations, treatment and associated factors for wound necrosis. The journal of venomous animals and toxins including tropical diseases 26, e20200043. https://doi.org/10.1590/1678-9199-JVATITD-2020-0043

Mao, Y. C., Liu, P. Y., Chiang, L. C., Lee, C. H., Lai, C. S., Lai, K. L., Lin, W. L., Su, H. Y., Ho, C. H., Doan, U. V., Maharani, T., Yang, Y. Y., & Yang, C. C. (2021). Clinical manifestations and treatments of Protobothrops mucrosquamatus bite and associated factors for wound necrosis and subsequent debridement and finger or toe amputation surgery. Clinical toxicology (Philadelphia, Pa.) 59(1), 28–37. https://doi.org/10.1080/15563650.2020.1762892

Su, H. Y., Huang, S. W., Mao, Y. C., Liu, M. W., Lee, K. H., Lai, P. F., & Tsai, M. J. (2018). Clinical and laboratory features distinguishing between Deinagkistrodon acutus and Daboia siamensis envenomation. The journal of venomous animals and toxins including tropical diseases 24, 43. https://doi.org/10.1186/s40409-018-0179-2

Cheng, C. L., Mao, Y. C., Liu, P. Y., Chiang, L. C., Liao, S. C., & Yang, C. C. (2017). Deinagkistrodon acutus envenomation: a report of three cases. The journal of venomous animals and toxins including tropical diseases 23, 20. https://doi.org/10.1186/s40409-017-0111-1

2. Present the statistical analysis in the results section.

Reply: Using the four parameter logistic curve-fitting in SigmaPlot (version 12.0; SYSTAT Software Inc., CA, USA), the statistical values (mean, standard error, and 95% confidence interval) for E_max_ and EC_50_ were determined. As the venom samples from each species are pooled from individual snakes, we refrained from testing differences among treatments using t-tests, ANOVA, or nonparametric statistics, as observed in other studies (as referenced below). We have described the limitations of our study at Line 439-443 and made revisions to the relevant text at Line 187-189, 259-262.

References:

Alsolaiss, J., Alomran, N., Hawkins, L., & Casewell, N. R. (2023). Commercial Antivenoms Exert Broad Paraspecific Immunological Binding and In Vitro Inhibition of Medically Important Bothrops Pit Viper Venoms. Toxins 15(1), 1. https://doi.org/10.3390/toxins15010001

Alomran, N.; Blundell, P.; Alsolaiss, J.; Crittenden, E.; Ainsworth, S.; Dawson, C.A.; Edge, R.J.; Hall, S.R.; Harrison, R.A.; Wilkinson, M.C.; et al. Exploring the Utility of Recombinant Snake Venom Serine Protease Toxins as Immunogens for Generating Experimental Snakebite Antivenoms. Toxins 2022, 14, 443. https://doi.org/10.3390/toxins14070443

Chaisakul, J., Rusmili, M. R. A., Alsolaiss, J., Albulescu, L. O., Harrison, R. A., Othman, I., & Casewell, N. R. (2020). In Vitro Immunological Cross-Reactivity of Thai Polyvalent and Monovalent Antivenoms with Asian Viper Venoms. Toxins, 12(12), 766. https://doi.org/10.3390/toxins12120766

Tan, C. H., Tan, K. Y., Lim, S. E., & Tan, N. H. (2015). Venomics of the beaked sea snake, Hydrophis schistosus: A minimalist toxin arsenal and its cross-neutralization by heterologous antivenoms. Journal of proteomics 126, 121–130. https://doi.org/10.1016/j.jprot.2015.05.035

Leong, P. K., Tan, C. H., Sim, S. M., Fung, S. Y., Sumana, K., Sitprija, V., & Tan, N. H. (2014). Cross neutralization of common Southeast Asian viperid venoms by a Thai polyvalent snake antivenom (Hemato Polyvalent Snake Antivenom). Acta tropica 132, 7–14. https://doi.org/10.1016/j.actatropica.2013.12.015

Harrison, R. A., Oluoch, G. O., Ainsworth, S., Alsolaiss, J., Bolton, F., Arias, A. S., Gutiérrez, J. M., Rowley, P., Kalya, S., Ozwara, H., & Casewell, N. R. (2017). Preclinical antivenom-efficacy testing reveals potentially disturbing deficiencies of snakebite treatment capability in East Africa. PLoS neglected tropical diseases 11(10), e0005969. https://doi.org/10.1371/journal.pntd.0005969

Tan, C. H., Liew, J. L., Tan, N. H., Ismail, A. K., Maharani, T., Khomvilai, S., & Sitprija, V. (2017). Cross reactivity and lethality neutralization of venoms of Indonesian Trimeresurus complex species by Thai Green Pit Viper Antivenom. Toxicon 140, 32–37. https://doi.org/10.1016/j.toxicon.2017.10.014

3. Why were very short dose intervals used in the LD50 assays (0.4-9 ug/g)?

Reply: In our LD_50_ assays, we opted for short dose intervals to ensure a high-resolution analysis of the toxic response. This finer dosing gradient was pivotal in accurately pinpointing the LD_50_ value, as it allowed for a detailed mapping of the dose-dependent mortality curve, which is critical for our toxicological assessment.

4. The discussion of this study could be improved by providing more data on snakebite accidents and linking them to the neutralization data obtained here.

Reply: To date, only two case reports are available indicating that patients bitten on the finger by *T. gracilis* not only experience local hemorrhagic blisters or tissue necrosis but also systemic coagulopathy, even after immediate administration of TsPmAV. We have included an experiment involving SDS-PAGE analysis of *T. gracilis* venom, comparing it to other venoms in our study. This addition aims to enhance the discussion regarding antivenom binding capacity levels and improve our understanding of toxins that may not be recognized by traditional antivenoms. In an ongoing study investigating the binding capacity of TsPmAV to *T. gracilis* venom through immunoaffinity column chromatography, we observed lower reactivity with P-I SVMPs and certain SVSPs/PLA_2_ when exposed to the antivenom (Tsai et al., unpublished data). Silva et al. (2021a, b) discovered that the antivenom targeting *Bothrops* venom has limitations in addressing local effects such as hemorrhage and neutralizing SVSPs. However, they observed that supplementing the antivenom with a serine protease inhibitor can enhance its overall efficacy. In subsequent studies, testing the neutralization efficacy by adding inhibitors specific to P-I SVMPs or SVSPs/PLA_2_ to the existing antivenom could be explored to enhance therapeutic success for both local and systemic symptoms. We have made revisions to the relevant text at Line 33-36, 112-114, 169-174, 200-209, 282-307, 395-420, 452-459.

References:

Silva, G. M., Berto, D. H., Lima, C. A., Waitman, K. B., Lima, C. F. G., Prezoto, B. C., Vieira, M. L., Rocha, M. M. T., Gonçalves, L. R. C., & Andrade, S. A. (2021a). Synergistic effect of serine protease inhibitors and a bothropic antivenom in reducing local hemorrhage and coagulopathy caused by *Bothrops jararaca* venom. Toxicon 199, 87–93. https://doi.org/10.1016/j.toxicon.2021.06.009

Silva, G. M., de Souza, D. H. B., Waitman, K. B., Ebram, M. C., Fessel, M. R., Zainescu, I. C., Portaro, F. C., Heras, M., & de Andrade, S. A. (2021b). Design, synthesis, and evaluation of *Bothrops* venom serine protease peptidic inhibitors. The Journal of Venomous Animals and Toxins Including Tropical Diseases 27, e20200066. https://doi.org/10.1590/1678-9199-JVATITD-2020-0066
